# M-CSF in a new biomarker panel with HE4 and CA 125 in the diagnostics of epithelial ovarian cancer patients

**DOI:** 10.1186/s13048-015-0153-3

**Published:** 2015-05-03

**Authors:** Grażyna Ewa Będkowska, Sławomir Ławicki, Ewa Gacuta, Przemysław Pawłowski, Maciej Szmitkowski

**Affiliations:** Department of Haematological Diagnostics, Medical University, Białystok, Poland; Department of Biochemical Diagnostics, Medical University Białystok, Waszyngtona 15A, Białystok, 15-269 Poland; Department of Perinatology, Medical University, Białystok, Poland; Department of Pediatric Ophthalmology with Squint Treatment Unit, Medical University, Białystok, Poland

**Keywords:** M-CSF, HE4, CA 125, Epithelial ovarian cancer, Tumor markers

## Abstract

**Background:**

We investigated plasma levels of M-CSF and conventional tumor markers (HE4 and CA 125) in epithelial ovarian cancer patients as compared to control groups: benign ovarian tumor patients (cysts) and healthy subjects.

**Methods:**

M-CSF levels were determined by ELISA, HE4 and CA 125 levels - by CMIA method.

**Results:**

Our results have demonstrated significant differences in the concentration levels of M-CSF, CA 125 and HE4 between the groups of ovarian cancer patients, cysts patients and the healthy controls. In the groups tested M-CSF demonstrated equal to or higher values than both CA 125 and HE4 in diagnostic sensitivity (SE), positive and negative predictive values (PPV, NPV), and in the area under the ROC curve (AUC), particularly in the group with the *serous epithelial* sub-type of OC. Moreover, CA 125 showed better results of the aforementioned diagnostic criteria than HE4. The combined use of the parameters studied resulted in a further, significant increase in the value of the diagnostic indicators and in the value of the diagnostic power (AUC), especially in the early stages of ovarian cancer.

**Conclusions:**

These findings suggest a high usefulness of M-CSF in diagnosing the *serous* sub-type of epithelial ovarian cancer and in discriminating between cancer and non-carcinoma lesions, particularly in new diagnostic panels in combination with CA 125 and HE4 for the detection of EOC in the early stages.

**Electronic supplementary material:**

The online version of this article (doi:10.1186/s13048-015-0153-3) contains supplementary material, which is available to authorized users.

## Background

Epithelial ovarian cancer (EOC) remains the most lethal type of all gynecological cancers despite the development of new treatments and therapies. The malignant tumor of the ovaries occurs at all ages with variation in histological sub-type according to age [1,2]. Established risk factors associated with the increasing prevalence of OC include genetics (BRCA1- breast cancer type 1 susceptibility protein and BRCA2 - breast cancer type 2 susceptibility protein), age (primarily perimenopausal and postmenopausal status), positive familial history (5–10%), diet (rich in meat and saturated fats), and other reproductive factors [1,3,4]. A lack of precise early warning signs is one of the factors that further contributes to the fact that only 25% of ovarian tumors are identified at a treatable stage I [3]. In the majority of cases OC is diagnosed in the late stages of the disease when patients have metastatic disease at presentation [2]. Detection at an early stage offers a potential reduction in mortality. Therefore, finding markers which would identify a malignant cell transformation as early as possible is of critical importance [5].

The established tumor markers, such as CA 125 (carbohydrate antigen 125) or human epididymis protein 4 (HE4), can be used in the diagnosis and monitoring of epithelial ovarian cancer [6-8]. A number of researchers and clinicians have been investigating many new diagnostic markers, some of which have recently shown promise, which may be useful in the diagnosis of this type of cancer [7,9]. Different types of substances, for example cytokines such as macrophage-colony stimulating factor (M-CSF), vascular endothelial growth factor (VEGF) and interleukin 10 (IL-10) [10-13], metalloproteinases (MMP-2, MMP-7, MMP-9) and the tissue inhibitor of metalloproteinase 1 (TIMP-1) [14-16] or proteomic biomarkers (mesothelin, B7-H4, DcR3, spondin-2) [17,18] are currently being investigated.

Macrophage-colony stimulating factor is one of the cytokines called hematopoietic growth factors (HGFs). M-CSF regulates the growth, differentiation and functionality of neutrophils or macrophages. Additionally, M-CSF has also been implicated in the pathogenesis of cancer disease [19,20]. A number of authors focused on the role of M-CSF and its receptor in epithelial malignancies, including those of breast [21-23], lung [24,25], pancreatic [26], cervical [27,28], and ovarian origin [29-31].

The aim of this study was to determine plasma levels of macrophage-colony stimulating factor in comparison to plasma levels of HE4 and the established CA 125 tumor marker in epithelial ovarian cancer (EOC) patients in relation to the control groups: patients with a benign ovarian tumor and healthy subjects. Additionally, comparisons between plasma levels of the parameters tested and cancer stage, its histological sub-type and histological type of benign ovarian tumors were performed. Furthermore, the diagnostic criteria (sensitivity, specificity, positive and negative predictive values) and the receiver-operating characteristic curve (ROC) for the cytokine tested (M-CSF), HE4 and CA 125 alone and in combinations were defined. Moreover, a correlation between the three parameters studied was established.

The data obtained may be used in the evaluation of M-CSF usefulness in diagnosing the stages and histological sub-types of ovarian cancer and in discriminating between ovarian cancer and benign ovarian tumors, especially when analysed with HE4 and CA 125.

## Methods

### Patients

Table 1 shows the characteristics of patients and control groups. The study included 110 epithelial ovarian cancer patients (*serous* and *endometrioid* sub-types) diagnosed by the Gynecology Group. The control groups comprised 70 benign ovarian tumor patients (*cystis serous* or *cystis endometrioides*) and 50 healthy volunteers. All participants enrolled in the study (cancer and control groups) had postmenopausal status at the time of blood collection. Clinical stages and histological classification based on the criteria of the International Federation of Gynecology and Obstetrics (FIGO) were established in all cases. The ovarian cancer patients and the control group (benign lesions) were treated in the Department of Gynecology, Białystok Medical University Teaching Hospital, Poland, between 2006–2012. Epithelial ovarian cancer and benign ovarian tumor histopathology was established in all cases. Patients with renal failure were excluded from the study due to significantly elevated HE4 concentration levels, indistinguishable from ovarian cancer. Written consent including participants’ own statements regarding their medical history (i.e. data related to reproductive history, personal or family history of cancer, general health issues - hospitalization or surgery, use of medication) and lifestyle habits including smoking was obtained from all the subjects.

**Table 1:** **Characteristics of ovarian cancer patients and control groups: benign ovarian tumor and healthy subjects**

**Study group**		**Number of patients**
Epithelial ovarian cancer patients		110 (100%)
• Median age (range)		58 (46–80)
-sub-type *serous epithelial*		61 (55%)
• Median age (range)		58 (46–81)
-sub-type *endometrioid epithelial*		49 (45%)
• Median age (range)		59 (48–86)
Tumor stage	IA	6 (5.4%)
	IB	8 (7.3%)
	IC	14 (12.7%)
	IIA	9 (8.2%)
	IIB	10 (9.1%)
	IIC	9 (8.2%)
	IIIA	10 (9.1%)
	IIIB	10 (9.1%)
	IIIC	8 (7.3%)
	IV (metastases)	26 (23.6%)
Menopausal status: - postmenopausal		110 (100%)
Benign ovarian tumor patients		70 (100%)
-type *cystis serous*		35 (50%)
-type *cystis endometrioides*		35 (50%)
Median age (range)		52 (48–68)
Menopausal status: - postmenopausal		70 (100%)
Healthy subjects		50 (100%)
Median age (range)		56 (48–66)
Menopausal status: - postmenopausal		50 (100%)

None of the patients had received chemo- or radiotherapy before blood sample collection. Pretreatment staging procedures included physical and blood examinations, ultrasound scanning and chest X-rays. In addition, CT (computed tomography) scans or MRI (magnetic resonance imaging) were performed where necessary.

Healthy patients were recruited from apparently healthy female employees of Białystok Medical University Teaching Hospital between 2006 and 2012. They were not referred from other medical centers. All subjects had undergone annual check-ups (laboratory tests, chest x-ray, cervical cytology screening, mammography). Subjects with a clinical history of prior endometriosis or mild gynecological conditions were excluded. Women included in the control group were volunteers who reported no prior history of gynecological conditions and displayed no visible or perceptible changes in the adnexa. The group were examined by a gynecologist prior to blood collection and an ultrasound examination was performed in every case.

The study was approved by the local Ethics Committee of the Medical University in Białystok, numbers: R-I-002/314/2009 and R-I-002/262/2010 and all the patients gave their informed consent for the participation in the study.

### Biochemical analyses

Venous blood samples were collected from every patient. Blood was collected into a heparin sodium tube, centrifuged 1000 rpm for 15 min. to obtain plasma samples, and stored at-85^0^ C until assayed. M-CSF was measured with the enzyme-linked immunosorbent assay (ELISA) (Quantikine Human HGFs Immunoassay; R & D systems, Abingdon, United Kingdom), according to the manufacturer’s protocols. Duplicate samples were assessed for each patient. The intra-assay coefficient of variation (CV%) of M-CSF - 3.4% at a mean concentration of 227 pg/ml, SD = 7.7. The inter-assay coefficient of variation (CV%) of M-CSF - 3.1% at a mean concentration of 232 pg/ml, SD = 7.3. The assay showed no significant cross-reactivity or interference with numerous human cytokines and other growth factors.

Plasma concentrations of HE4 and CA 125 were measured by chemiluminescent microparticle immunoassay (CMIA) (Abbott, Chicago, IL, USA). The intra-assay CV for HE4 - 3.7% at a mean concentration of 39.0 pmol/L, SD = 1.4. The inter-assay CV for HE4-2.8% at a mean concentration of 39.0 pmol/L, SD = 1.1. The intra-assay CV for CA 125 is reported to be 2.4% at a mean concentration of 43.5 U/ml, SD = 1.1. The inter-assay CV for CA 125 is reported to be 3.9% at a mean concentration of 43.5 U/ml, SD = 1.7.

### Statistical analysis

The statistical analysis was performed using the STATISTICA 8.0 PL program. A preliminary statistical analysis (Chi-square test) revealed that the distribution of cytokine and tumor marker levels did not follow normal distribution. Consequently, nonparametric methods were used to compare levels of the parameters tested between the groups of patients. Comparisons between two groups were performed using the Mann–Whitney test, between multiple groups Kruskal-Wallis tests were calculated with post hoc comparisons according to Dwass-Steele-Critchlow-Fligner method. The ROC analyses were utilized in the evaluation of the diagnostic power of tumor markers and the construction of the curves was performed using GraphRoc Program for Windows. Markers were compared by assessing the significance of differences between the areas under their corresponding ROC curves. The *cut-off* points of M-CSF (575.80 pg/ml), HE4 (75.90 pmol/L) and CA 125 (28.40 U/ml) were calculated as 95th percentile from the control group of healthy blood donors.

Data were presented as median and range. Statistically significant differences were defined as comparisons resulting in p < 0.05. The Spearman rank correlation was used in the correlation analyses.

## Results

The median of M-CSF levels, similarly to the median levels of the comparative tumor markers HE4 and CA 125 in the total group of OC and in every stage of advancement (I-IV) of cancer disease were significantly higher when compared to the healthy controls (p < 0.001 in all cases) (Table 2). Moreover, plasma concentrations of all tested parameters were significantly higher in more advanced stages (III-IV) than those found in the early stages (I-II) - p < 0.001 in all cases.

**Table 2:** **Plasma levels of M-CSF, CA 125 and HE4 in tested groups**

	**Groups**		**M-CSF**	**HE4**	**CA 125**
			**(pg/ml)**	**(pmol/L)**	**(U/ml)**
**Ovarian cancer** Median Range	**stage I**		^**1**^	^**1/2**^	^**1**^
			444.40	83.54	63.62
			159.80 -2702.20	27.50-1093.80	12.50-650.4 0
	**stage II**		^**1/2**^	^**1/2**^	^**1/2**^
			619.76	62.64	61.62
			221.70-1764.00	24.00-625.10	8.40-998.00
	**stage III**		^**1/2/4**^	^**1/2/4**^	^**1/2/4**^
			706.45	117.92	766.84
			200.05-3791.05	47.00-1500.00	9.84-2060.78
	**stage IV**		^**1/2/4**^	^**1/2/4**^	^**1/2/4**^
			1009.40	198.14	531.92
			235.64-2091.75	36.40-1944.20	13.64-8602.30
	**Total group**		^1/2^	^**1/2**^	^**1/2**^
			633.00	103.64	133.39
			159.80-3791.05	24.00-1944.20	8.40-8602.30
	***Serous epithelial***		^**1/5/6/7**^	^**1/5/6/7**^	^**1/5/6/7**^
			794.05	126.24	171.24
			221.7-3791.05	28.60-1944.20	8.40-8602.30
	***Endometrioid epithelial***		^**1/6**^	^**1/6/7**^	^**1/6/7**^
			606.48	68.50	114.24
			159.80-3396.20	24.00-1740.00	10.92-1425.00
**Control groups** Median Range	**Benign ovarian tumor**	***Cystis endometrioides***	^**3**^	^**3**^	^**3/8**^
			434.28	23.18	43.44
			125.30-2209.30	14.00-68.60	7.50-2748.00
		***Cystis serous***	^**3**^	^**8**^	^**3**^
			468.35	43.34	20.69
			166.90-1604.40	25.40-159.94	5.40-451.80
		**Total group**	^**3**^		^**3**^
			448.10	42.60	27.74
			125.30-2209.30	14.00-159.94	5.40-2748.00
	**Healthy subjects**		298.55	44.32	10.02
			119.63-1097.00	6.20-122.30	5.06-36.60

^**1**^statistically significant when comparing EOC patients with healthy subjects.

^**2**^statistically significant when comparing EOC patients with benign ovarian tumor total group.

^**3**^statistically significant when comparing patients with benign ovarian tumor and healthy subjects.

^**4**^statistically significant when comparing EOC patients in stage III or IV with stage I or II.

^**5**^statistically significant when comparing EOC patients i.e. sub-type *serous* with sub-type *endometrioid*.

^**6**^statistically significant when comparing with benign ovarian tumor group i.e. type *cystis endometrioides*.

^**7**^statistically significant when comparing with benign ovarian tumor group i.e. type *cystis serous*.

^**8**^statistically significant when comparing patients with benign ovarian tumor i.e. type *cystis endometrioides* with type *cystis serous*.

Patients with ovarian cancer (total group) had statistically considerably higher median levels of the parameters researched (p < 0.001 in all cases) than those observed in the group with benign ovarian tumors (Table 2). We also noticed significantly higher concentrations of M-CSF in II-IV (II - p = 0.045; III-IV - p < 0.001), of HE4 in I-IV (I - p = 0.018; II - p = 0.001; III-IV - p < 0.001) and of CA 125 in stages II-IV (II - p = 0.034; III-IV - p < 0.001) of EOC than in the control group with nonmalignant lesions of the ovary.

In the case of the total group with benign ovarian tumors, the concentrations of M-CSF and CA 125 were significantly different than in healthy subjects (p < 0.001).

The analysis according to the histopathological sub-types of EOC revealed statistical differences in the concentrations of M-CSF, HE4 and CA 125 between every cancer sub-type group (*serous* and *endometrioid*) and benign tumors control group (p < 0.001 in all cases). It was also observed that the distribution of all the tested parameters among two histological sub-types of epithelial ovarian cancer were significantly different (p = 0.012; p = 0.014; p < 0.001; respectively) (Table 2).

Plasma levels of M-CSF and CA 125 in the groups with *cystis endometrioides* and *cystis serous* were higher than those in healthy women (in all cases p < 0.001). Interestingly, the plasma level of HE4 was significantly lower in patients with *cystis endometrioides* in comparison to the healthy controls (p = 0.042). In addition, we noticed significant differences in the median HE4 (p = 0.046) and CA 125 levels (p = 0.039) when comparing the *cystis endometrioides* to the *cystis serous* group (Table 2).

The Spearman’s rank correlation was used in the analysis of dependence between the investigated parameters (Additional file 1: the Spearman’s rank correlation–results, data not shown). Our analysis revealed positive correlations between the HE4 and CA 125 concentrations in the total group of EOC (R = 0.47, p < 0.001), between the M-CSF and CA 125 (R = 0.4, p = 0.046) in patients with stage II cancer as well as between the HE4 and CA 125 levels according to the histopathological sub-types of EOC: *endometrioid* (R = 0.31, p = 0.037) or *serous* (R = 0.35, p = 0.008). Moreover, positive correlations were also observed between the M-CSF and CA 125 levels in the healthy control group (R = 0.32, p = 0.026). The single negative correlation was obtained for the HE4 and CA 125 concentrations (R = -0.41, p = 0.036) in patients with stage II EOC.

Table 3 shows the diagnostic criteria of parameters tested: sensitivity (SE), specificity (SP), positive predictive value (PPV) or negative predictive value (NPV) in the EOC patients. In the total group of EOC patients M-CSF presented the highest diagnostic sensitivity (70%). HE4 had the highest diagnostic SE in stage I of the disease from among all the parameters tested, although M-CSF demonstrated better results in stages II-IV. We also observed a further increase in the SE value in more advanced cancer stages (with the exception of HE4 in stage II). The combined use of the parameters tested resulted in a dramatic increase in the diagnostic SE for the combination of HE4 + CA 125 in stage I and II, M-CSF + CA 125 in stage III and of M-CSF + HE4 in stage IV of the disease. The maximum range of SE was obtained for the combination of all the parameters tested: in stage I - 80%, stage II- 92%, stage III- 96%, IV - 100% and in the total group of OC - 86%.

**Table 3:** **The diagnostic criteria of M-CSF and in combination with HE4 and CA 125 in epithelial ovarian cancer patients**

**Epithelial ovarian cancer**	**Diagnostic criteria (%)**	**M-CSF**	**HE4**	**CA 125**	**M-CSF+ HE4**	**M-CSF + CA 125**	**HE4+ CA 125**	**M-CSF + HE4 + CA 125**
**stage I**	SE	40	46	40	64	60	73	80
	SP	94	94	92	90	90	88	86
	PPV	87	88	87	86	86	86	85
	NPV	61	63	61	71	69	76	74
**stage II**	SE	68	29	63	80	80	81	92
	SP	94	94	92	90	90	88	86
	PPV	92	83	89	89	89	87	87
	NPV	75	57	72	82	82	82	91
**stage III**	SE	84	66	80	92	94	86	96
	SP	94	94	92	90	90	88	86
	PPV	93	92	91	90	90	86	87
	NPV	85	73	82	92	94	79	94
**stage IV**	SE	88	81	84	100	96	92	100
	SP	94	94	92	90	90	88	86
	PPV	93	93	91	91	91	88	88
	NPV	87	84	85	100	96	92	100
**Total group**	SE	70	55	66	84	83	84	86
	SP	94	94	92	90	90	88	86
	PPV	81	90	79	89	89	88	86
	NPV	76	68	73	85	84	86	86
***Serous epithelial***	SE	74	70	64	92	82	86	96
	SP	94	94	92	90	90	88	86
	PPV	92	92	89	90	80	79	87
	NPV	78	77	73	92	83	86	96
***Endometrioid epithelial***	SE	65	37	71	82	89	82	91
	SP	94	94	92	90	90	88	86
	PPV	91	86	90	89	82	87	87
	NPV	73	59	81	83	89	84	92

In regard to the histopathological sub-types of EOC, we demonstrated the highest range of diagnostic SE for M-CSF in the *serous epithelial* group and for CA 125 in the *endometrioid epithelial* group. Similarly, we observed an increase in the SE value in during the combined analysis of two and three parameters for both sub-types of EOC.

M-CSF and HE4 presented the highest diagnostic specificity values (94%) in every cancer group compared and in every histopathological sub-type of EOC. The combined use of the parameters studied resulted in a decrease in the diagnostic SP (Table 3).

In the total group of EOC patients the PPV had the highest values for HE4 (90%). HE4 reached the highest values of PPV in stage I in contrast to M-CSF, which reached the highest values in stages II-IV. Similarly, the combined use of the studied markers resulted in a decrease in the PPV values (Table 3). Additionally, we observed the highest PPV value for M-CSF and HE4 in the *serous* and for M-CSF in the *endometrioid* sub-types of EOC.

The negative predictive value in the total group of ovarian cancer reached the highest values for M-CSF (76%). M-CSF also obtained the highest value in stages II-IV with the exception of stage I, where HE4 consistently achieved the best results. Additionally, the NPV values showed a further increase during the combined analysis of the parameters tested (Table 3). The maximum value of NPV was obtained for M-CSF + HE4 and for M-CSF + HE4+ CA 125 in stage IV of the disease although very high values were also observed for the combination of two (69–94%) and three (74–94%) biomarkers together in stages I-III of EOC. M-CSF had the highest values in the group with *serous* and CA 125 in the group with *endometrioid* sub-types of OC (Table 3).

**Figure 1:**
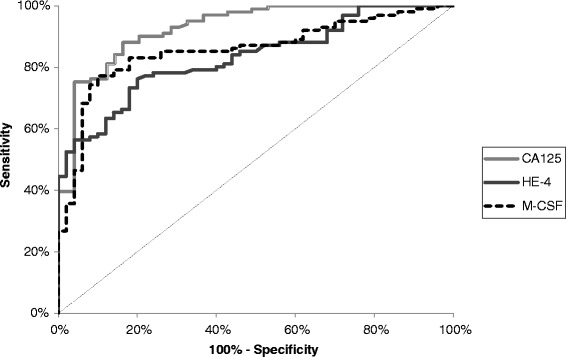
Diagnostic criteria of ROC curve for M-CSF, HE4 and CA 125 in total OC tested group.

**Figure 2:**
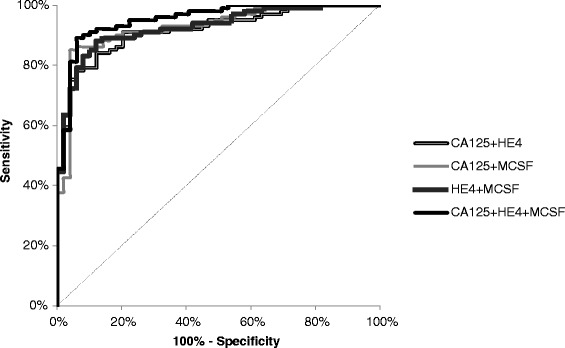
Diagnostic criteria of ROC curve for M-CSF, HE4 and CA 125 in combined analysis in total OC tested group.

The relationship between the diagnostic sensitivity and specificity was illustrated by the ROC curve. Table 4 shows the results of our in-depth analysis of the AUC (area under the ROC curve) of every biomarker, alone and in combination, for every stage and for every investigated histological sub-type of the disease. The CA 125 area (0.9277) under the ROC curve is the largest in the total group of EOC patients and its value was marginally higher than that of M-CSF or HE4 (Figures 1 and 2). The AUC of CA 125 was also the largest in the group of patients with stage IV, but interestingly the AUC of M-CSF was the largest in the group with stages I-III of the disease. The area under the ROC curve of all the biomarkers tested clearly illustrates an increase in their diagnostic power concurrent with the stage of advancement of the disease (with the exception of HE4 and M-CSF in stage IV) (Table 4). Additionally, the combination of all the parameters resulted in an increase in the diagnostic power in every case to the value of: 0.9069 in stage I, 0.9443 in stage II, 0.9714 in stage III and 0.9529 in the total group of EOC. Repeatedly, the largest area under the ROC curve was indicated for the combination of all the aforementioned markers in patients with stage IV cancer. According to histopathological classification, CA125 achieved the best result in *endometrioid epithelial* (0.9129) and M-CSF in *serous epithelial* sub-types of ovarian cancer (0.9058). The AUCs of M-CSF and the comparative tumor markers were significantly higher compared to AUC = 0.5 in every group of EOC studied (in all cases p < 0.001).

**Table 4:** **The diagnostic criteria of the ROC curve for M-CSF and in combination with HE4 and CA 125 in epithelial ovarian cancer patients**

**Epithelial ovarian cancer**	**The ROC criteria**	**M-CSF**	**HE4**	**CA 125**	**M-CSF+ HE4**	**M-CSF+ CA 125**	**HE4 + CA 125**	**M-CSF+ HE4 + CA 125**
**stage I**	AUC	0.7676*	0.7285*	0.7653*	0.8536*	0.8702*	0.8326*	0.9069*
	SE	0.0681	0.0708	0.0416	0.0496	0.0433	0.0538	0.0353
	95% C.I.	0.604-0.871	0.590-0.867	0.692-0.794	0.756-0.951	0.785-0.955	0.728-0.938	0.838-0.976
	*p* AUC = 0.5	**<0.001**	**0.0013**	**0.0005**	**<0.001**	**<0.001**	**<0.001**	**<0.001**
**stage II**	AUC	0.8662*	0.7647*	0.8653*	0.9008*	0.9113*	0.9208*	0.9443*
	SE	0.0483	0.0562	0.0389	0.0387	0.0393	0.0314	0.0265
	95% C.I.	0.772-0.961	0.654-0.876	0.814-0.968	0.825-0.977	0.834-0.988	0.859-0.983	0.892-0.966
	*p* AUC = 0.5	**<0.001**	**<0.001**	**<0.001**	**<0.001**	**<0.001**	**<0.001**	**<0.001**
**stage III**	AUC	0.9336*	0.9257*	0.9114*	0.9624*	0.9608*	0.9764*	0.9714*
	SE	0.0317	0.0291	0.0428	0.0250	0.0302	0.0302	0.0222
	95% C.I.	0.871-0.996	0.869-0.984	0.828-0.995	0.913-1.011	0.902-1.020	0.903-1.020	0.928-1.015
	*p* AUC = 0.5	**<0.001**	**<0.001**	**<0.001**	**<0.001**	**<0.001**	**<0.001**	**<0.001**
**stage IV**	AUC	0.9096*	0.9125*	0.9474*	0.9872*	0.9665*	0.9422*	0.9894*
	SE	0.0414	0.0428	0.0260	0.0086	0.0214	0.0353	0.0083
	95% C.I.	0.829-0.991	0.829-0.996	0.897-0.998	0.970-1.004	0.925-1.008	0.873-1.012	0.973-1.006
	*p* AUC = 0.5	**<0.001**	**<0.001**	**<0.001**	**<0.001**	**<0.001**	**<0.001**	**<0.001**
**Total group**	AUC	0.8562*	0.8324*	0.9277*	0.9257*	0.9271*	0.9182*	0.9529*
	SE	0.0316	0.0321	0.0221	0.0212	0.0225	0.0223	0.0172
	95% C.I.	0.794-0.918	0.769-0.895	0.884-0.972	0.884-0.967	0.883-0.971	0.874-0.963	0.919-0.967
	*p* AUC = 0.5	**<0.001**	**<0.001**	**<0.001**	**<0.001**	**<0.001**	**<0.001**	**<0.001**
***Serous epithelial***	AUC	0.9058*	0.8722*	0.9046*	0.9491*	0.9558*	0.9390*	0.9659*
	SE	0.0306	0.0347	0.0301	0.0199	0.0199	0.0233	0.0160
	95% C.I.	0.846-0.966	0.805-0.964	0.846-0.964	0.910-0.989	0.917-0.995	0.893-0.985	0.930-0.997
	*p* AUC = 0.5	**<0.001**	**<0.001**	**<0.001**	**<0.001**	**<0.001**	**<0.001**	**<0.001**
***Endome-trioid epithelial***	AUC	0.7983*	0.7845*	0.9129*	0.8978*	0.8926*	0.8927*	0.9374*
	SE	0.0491	0.0467	0.0305	0.0331	0.0343	0.0330	0.0241
	95% C.I.	0.702-0.894	0.694-0.876	0.854-0.973	0.833-0.963	0.825-0.960	0.828-0.958	0.890-0.985
	*p* AUC = 0.5	**<0.001**	**<0.001**	**<0.001**	**<0.001**	**<0.001**	**<0.001**	**<0.001**

(C.I. – confidence intervals of AUC).

*statistically significant when comparing tested parameters AUC’s with 0.5 AUC.

## Discussion

The search for an effective screening test for ovarian cancer has been the focus of intensive research efforts. Blood flows into and out of tumors and circulates tumor-specific protein profiles, making serum or plasma the ideal biological media for finding a screening biomarker [13]. The co-expression of M-CSF and its transmembrane tyrosine kinase receptor has been detected in epithelial ovarian carcinoma and could be involved in the autocrine growth stimulation of this type of cancer. M-CSF, known as a colony stimulating factor 1 (CSF-1), is also a potent chemoattractant for monocytes, which in turn, can produce factors that stimulate proliferation of ovarian tumor cells including interleukin-1 (IL-1), interleukin-6 (IL-6) and tumor necrosis factor (TNF). Clinical specimens from ovarian cancer metastases display strong immunostaining for both CSF-1 and its receptor in contrast to noninvasive borderline tumors and to benign ovarian tissue [27,29]. HE4 is a novel protein and one of the more promising biomarkers for improving the diagnostic performance in ovarian cancer detection. This is a precursor to the epididymal secretory protein E4. It is overexpressed in ovarian carcinomas, but there is minimal expression in normal ovarian tissue. HE4 promotes migration and adhesion of ovarian cancer cells. It was reported that HE4 could be used as a biomarker for ovarian cancer with a specificity higher than that of CA 125. This protein was particularly highly expressed in histologic subtypes of *serous* or *endometrioid* ovarian carcinoma [6-8].

In this study we investigated the diagnostic usefulness of M-CSF used alone and in combination with HE4 and CA 125 in patients with EOC before surgical intervention or chemo- and/or radiotherapy. Furthermore, we estimated the diagnostic utility of these biomarkers in correlation to the stage and histological type of ovarian cancer.

Our results show that M-CSF as well as HE4 and CA 125 plasma levels in the total group of ovarian cancer patients were statistically significantly higher in comparison to the group of healthy controls. Data regarding M-CSF obtained in the present study are also in agreement with our previous studies [10] and with research results of other authors who compared ovarian cancer patients with healthy volunteers, although there were differences in the number and the composition of the groups tested [32-34]. Significantly elevated levels of M-CSF have also been found in the sera of patients with malignancies of the reproductive organs [32,35,36], breast [37] or pancreatic and ampullary cancer [26]. Moreover, it was observed that M-CSF concentrations were statistically different in every group (the analysis related to the stage of advancement of OC) compared to the healthy subjects. Similar results for M-CSF were confirmed in our previous investigations [10] and in the studies of other authors [34], although their observations regarding the highest concentrations of M-CSF related to the early stages of the disease and concerned a larger number of sub-types of EOC than our current study (*serous*, *endometrioid*, *mucinous*, *clear cell*). It is worth noting that Suzuki and colleagues [38] found no significant difference in M-CSF levels between early and advanced stages of OC, and this is in opposition to the results of our study in which a group of postmenopausal women was studied [10]. It should also be emphasized that M-CSF and its receptor gene, *c-fms*, were expressed in gynecological malignancies, and the co-expression of M-CSF and *c-fms* might be related to progression to the metastatic state [27] and is associated with poor prognosis [30,38]. Furthermore, it was observed that 56% (14/25) of the patients with clinically evident OC and normal levels of CA125 had elevated levels of M-CSF [39]. Our results are consistent with the results of other authors who observed increased levels of HE4 and CA 125 in the group of ovarian cancer patients compared to the healthy women group [8,40], although the groups tested were far smaller (60 and 30 women respectively). Similar results were obtained by Molina and others [41] who observed higher concentrations of the aforementioned tumor markers in ovarian (111 patients) as well as in other active gynecological cancers (32 patients) or in different benign gynecological lesions (285 subjects). Furthermore, other researchers have found circulating concentrations of HE4 and CA 125 significantly higher in patients with early and late stages of cancer compared with healthy women, although there were differences in ethnical characteristics of the population selected [42,43]. The analysis of research results published by other authors has revealed a finding, almost identical to ours, regarding significant differences in concentration levels of both tumor markers which were clearly related to the stage of the disease, with significantly higher concentration levels in the advanced stages III-IV than in stages I-II [41,44].

In line with our previous report [10] and others publications [32,41,45] there was a difference in M-CSF concentrations between the EOC patients and the benign (cysts) tumors groups. Gaducci *et al.* [45] discovered that preoperative serum M-CSF levels were significantly elevated in patients with epithelial OC when compared to those with a benign ovarian disease (cases of *serous*, *mucinous* and *endometriotic cysts* and also of *fibromas*, *thecoma* and benign *cystic teratomas* were analysed). Suzuki *et al.* [38] confirmed these findings, although malignant germ cell tumors of the ovary and mature *cystic teratomas* were studied. Furthermore, Burke *et al.* [46] revealed mRNA expression for M-CSF and its receptor in a majority of malignant and benign ovarian biopsies with minimal to no expression in the normal epithelium. Takagi *et al.* [32] observed serum M-CSF levels in this type of cancer significantly different from those in patients with a benign ovarian tumor (p < 0.01) and with leiomyoma (p < 0.001) as well as between endometrial (stages Ib-III) and cervical (stages III-IV) cancer patients and patients with leiomyoma (p < 0.05). Comparable results were obtained in studies conducted on patients with breast malignancies [37]. The findings of the current study regarding comparative tumor markers correspond to the findings reported in the existing literature [43,47,48] and to our previous publications [10,36]. Significantly higher serum concentrations of HE4 and CA 125 (p = 0.005, p = 0.001, p < 0.0001, p < 0.001, respectively) [6,41,49,50] were found in OC patients than in those with benign diseases, although there were differences in the menopausal status and composition of the groups compared. In addition, HE4 showed a greater than CA 125, satisfactory ability to distinguish ovarian cancer from endometriosis [51].

In contrast to the results published by Suzuki *et al.* [29] we observed significantly higher plasma levels of M-CSF in patients with a benign ovarian tumor than in healthy controls. These findings are similar to the results obtained in our previous studies conducted on female patients with uterine myoma [36]. Our present observations concerning both tumor markers are in agreement with published evidence, although HE4 is reported to be less frequently elevated than CA 125 in benign gynecologic disorders [8,52,53]. Furthermore, patients with *serous epithelial* OC displayed significantly different concentrations of all the parameters studied than those with *endometrioid epithelial* OC. We also demonstrated significant differences in the concentrations of the parameters tested in every group of OC sub-types vs patients with *cystis endometrioides* and with *cystis serous.* Comparable results, but only for CA 125, were published by Nolen *et al.* [54], although benign cases included a broad spectrum of non-malignant lesions representing a variety of histological origins. Significant differences were also observed, but only for HE4 and CA 125, between the patients with *cystis endometrioides* and *cystis serous*. These data are at variance with other publications [28], and may have been partly influenced by study participant selection, although similar results have recently been reported [7,41,55,56]. Unfortunately, we could not confirm our findings regarding M-CSF in other publications, since no reports on the subject are available.

The correlation between M-CSF, HE4 and CA 125 levels was estimated by Spearman’s rank correlation test and most of the measured values tended to increase for every marker. However, the degree of the correlation was not particularly strong (R: 0.32–0.47), and there were some discordant results. This suggests that each marker was elevated concurrently or under different conditions, and these results also support the necessity of combining the three markers. We found similar results in the existing literature [8,10,52].

In our analysis of postmenopausal subjects, we found that M-CSF alone provided the highest SE, PPV and NPV, (68–88%, 91–93%, 75–87% respectively) of any individual biomarker tested (with the exception of HE4 - stage I and CA 125 - EOC *endometrioid* sub-type). Our results are in agreement with the investigation of Skates *et al.* [33], whose study group comprised only 60 patients - 50% fewer than our study group, and with the publications of other authors [57] as well as with our previous studies [10,58]. The diagnostic SP was very high and reached the value of 94% for M-CSF and HE4, and 92% for CA 125 and these results are in accordance with [36,59] or at variance with [10] our earlier papers and the existing literature [41,52] but they may have been partly influenced by study participant selection.

Nonetheless, a multi-marker approach appears to hold promise for detecting early stage ovarian cancers [6]. Despite a decrease in specificity (86%) and PPV (85%), when M-CSF was combined with conventional markers, sensitivity levels improved to 80% - stage I, to 92–100% in stages II-IV and to 91% or 96% in every histological sub-type of OC. Moreover, in the present study we obtained a better outcome for the diagnostic SE than that attained by Skates *et al.* [33] and *Zhang et al.* [34] who found that multiple-marker panels (M-CSF, CA72-4 (carbohydrate antigen 72–4) and CA 125 - 70%; M-CSF, CA72-4, CA 125 and CA15-3 (carbohydrate antigen 15–3) - 71% respectively) significantly increased preoperative early-stage sensitivity. A comparable increase in the diagnostic SE was previously observed, although it regarded the combined analysis of VEGF with CA 125 and HE4 [59]. Similarly, NPV levels were improved to 74% - stage I, to 91–100% in stages II-IV and to 96% or 92% in the *serous* and *endometrioid* sub-types, substantially higher than NPV levels provided by either marker used alone. These observations are in opposition to the results of other authors who observed distinctly higher values of these diagnostic criteria for HE4 than for CA 125 in premenopausal subjects [60], or higher values of NPV for HE4 or CA 125 alone (72% and 92%, respectively) in a group of Asian women [61]. We were unable to compare our findings regarding the aforementioned panel of biomarkers with the findings of other authors, since no reports on the subject are available. The results of the current study support our previous findings regarding the usefulness of M-CSF and CA 125 in the diagnostics of this malignancy [10].

The AUC numerically describes the overall performance of a marker, with the AUC of 1 indicating a perfect SE and SP. We observed that M-CSF had the highest, comparable to conventional markers, diagnostic power in the groups with stages I-III of OC (0.7676, 0.8662, 0.9336, respectively) and in the patients with *serous* sub-type of EOC (0.9058). We demonstrated that the utilization of a combination of all the markers under investigation consistently provided the highest sensitivity for the detection of every stage, and particularly early stages, of EOC with the AUC value of 0.9069–0.9894, which was superior to the value of either marker used alone. Furthermore, we observed that the areas under the curve for the cytokine tested and the comparative markers were statistically significantly larger compared to AUC = 0,5 - borderline of diagnostic usefulness of the test. Test data published by Zhang *et al.* [34] are at variance with our present results (AUC for: CA 125–0.908, M-CSF - 0.792), although in a few previous studies the AUC values of CA 125 and HE4 for differentiating EOC were 0.82–0.95 and 0.85–0.96, respectively [6,49,50,52,56,62] and were significantly higher when the markers were used in combination [50], the results similar to our findings.

## Conclusions

In conclusion, challenges regarding the early diagnosis of epithelial ovarian cancer remain. Although hundreds of biomarkers have been identified as being associated with this common type of cancer, their efficacy in the early diagnosis of the disease has not yet been established. To our knowledge, this is the first study in which plasma levels of M-CSF, HE4 and CA 125 were simultaneously analysed in a relatively large group of untreated patients with primary malignant and nonmalignant ovarian tumors. While each marker has its own strengths and weaknesses, CA 125 still remains better than HE4 in the diagnostics of EOC patients. However, M-CSF obtained even better results than CA 125. Our findings suggest a highly potential role for M-CSF as a tumor marker in the diagnosis of the *serous* sub-type of EOC, particularly in combination with HE4 and CA 125 as a new diagnostic panel. The utilization of the tested parameters in combination may also measurably improve the OC diagnosis in the early stages. Additional studies with larger, early-stage carcinoma patient groups may be needed to further strengthen the statistical power of the evidence.

## Additional file

Additional file 1:
**The Spearman’s rank correlation–results.**

## Abbreviations

AUC: Area under ROC curve
BRCA1: Breast cancer type 1 susceptibility protein
BRCA2: Breast cancer type 2 susceptibility protein
CA 125: Carbohydrate antigen 125
CA 15–3: Carbohydrate antigen 15–3
CA 72–4: Carbohydrate antigen 72–4
CEA: Carcinoembryonic antygen
CMIA: Chemiluminescent microparticle immunoassay
CSF-1: Colony stimulating factor 1
CT: Computed tomography
DcR3 ELISA: Enzyme-linked immunosorbent assay
FIGO: International Federation of Gynecology and Obstetrics
HE4: Human epididymis protein 4
HGFs: Hematopoietic growth factors
IL-1: Interleukin 1
IL-6: Interleukin 6
IL-10: Interleukin 10
M-CSF: Macrophage-colony stimulating factor
MMP-2: Metalloproteinase 2
MMP-7: Metalloproteinase 7
MMP-9: Metalloproteinase 9
MRI: Magnetic resonance imaging
OC: Ovarian cancer
PPV: Positive predictive value
NPV: Negative predictive value
ROC: Receiver-operating characteristics
SE: Diagnostic sensitivity
SP: Diagnostic specificity
TNF: Tumor necrosis factor
TIMP-1: Tissue inhibitor of metalloproteinase 1
TNM: Tumor-nodulus-metastasis classification
VEGF: Vascular endothelial growth factor
